# Import and Export of Misfolded α-Synuclein

**DOI:** 10.3389/fnins.2018.00344

**Published:** 2018-05-23

**Authors:** Lilia Rodriguez, Maria M. Marano, Anurag Tandon

**Affiliations:** ^1^Tanz Centre for Research in Neurodegenerative Diseases, Krembil Discovery Tower, Toronto, ON, Canada; ^2^Department of Medicine, University of Toronto, Toronto, ON, Canada

**Keywords:** synucleinopathy, Parkinson disease, protein misfolding, proteostasis, protein spreading, endocytosis, fibrils, oligomers

## Abstract

In Parkinson's disease, intracellular α-synuclein (α-syn) inclusions form in neurons and are referred to as Lewy bodies. These aggregates spread through the brain following a specific pattern leading to the hypothesis that neuron-to-neuron transfer is critical for the propagation of Lewy body pathology. Here we review recent studies employing pre-formed fibrils generated from recombinant α-syn to evaluate the uptake, trafficking, and release of α-syn fibrils. We outline methods of internalization as well as cell surface receptors that have been described in the literature as regulating α-syn fibril uptake. Pharmacological and genetic studies indicate endocytosis is the primary method of α-syn internalization. Once α-syn fibrils have crossed the plasma membrane they are typically trafficked through the endo-lysosomal system with autophagy acting as the dominant method of α-syn clearance. Interestingly, both chaperone-mediated autophagy and macroautophagy have been implicated in the degradation of α-syn, although it remains unclear which system is chiefly responsible for the removal of α-syn fibrils. The major hallmark of α-syn spreading is the templating of misfolded properties onto healthy protein resulting in a conformational change; we summarize the evidence indicating misfolded α-syn can seed endogenous α-syn to form new aggregates. Finally, recent studies demonstrate that cells release misfolded and aggregated α-syn and that these processes may involve different chaperones. Nonetheless, the exact mechanism for the release of fibrillar α-syn remains unclear. This review highlights what is known, and what requires further clarification, regarding each step of α-syn transmission.

## Cell surface binding and internalization of α-synuclein fibrils

The hypothesis that α-synuclein (α-syn) pathology is propagated along neuronal pathways and by intercellular exchange implies at least three physiological processes: membrane binding and internalization by recipient cells, interaction with intracellular α-syn, and eventual secretion or transport into adjacent cells. There is now significant experimental evidence using *in vitro*-generated α-syn assemblies indicating that each of these steps can be observed in cell and animal models. For example, fibrils are efficiently internalized by cultured neuroblastoma cells and primary neurons without the need of transfection reagents (Lee et al., 2008a,b; Volpicelli-Daley et al., 2011). Similarly, α-syn monomers, oligomers, and fibrils injected into the murine olfactory bulb (OB) are rapidly internalized by neurons and glia that project to central olfactory structures in the brain (Rey et al., 2013, 2016). Uptake efficiency may also be cell-type dependent, as astrocytes appear to be more competent at internalizing α-syn fibrils compared to primary neurons (Loria et al., 2017). Moreover, microfluidic chambers have been used to characterize α-syn uptake in axons, dendrites, and the soma of neurons indicating that α-syn can be internalized through the plasma membrane in all cell compartments (Volpicelli-Daley et al., 2011; Freundt et al., 2012; Brahic et al., 2016).

**Figure 1:**
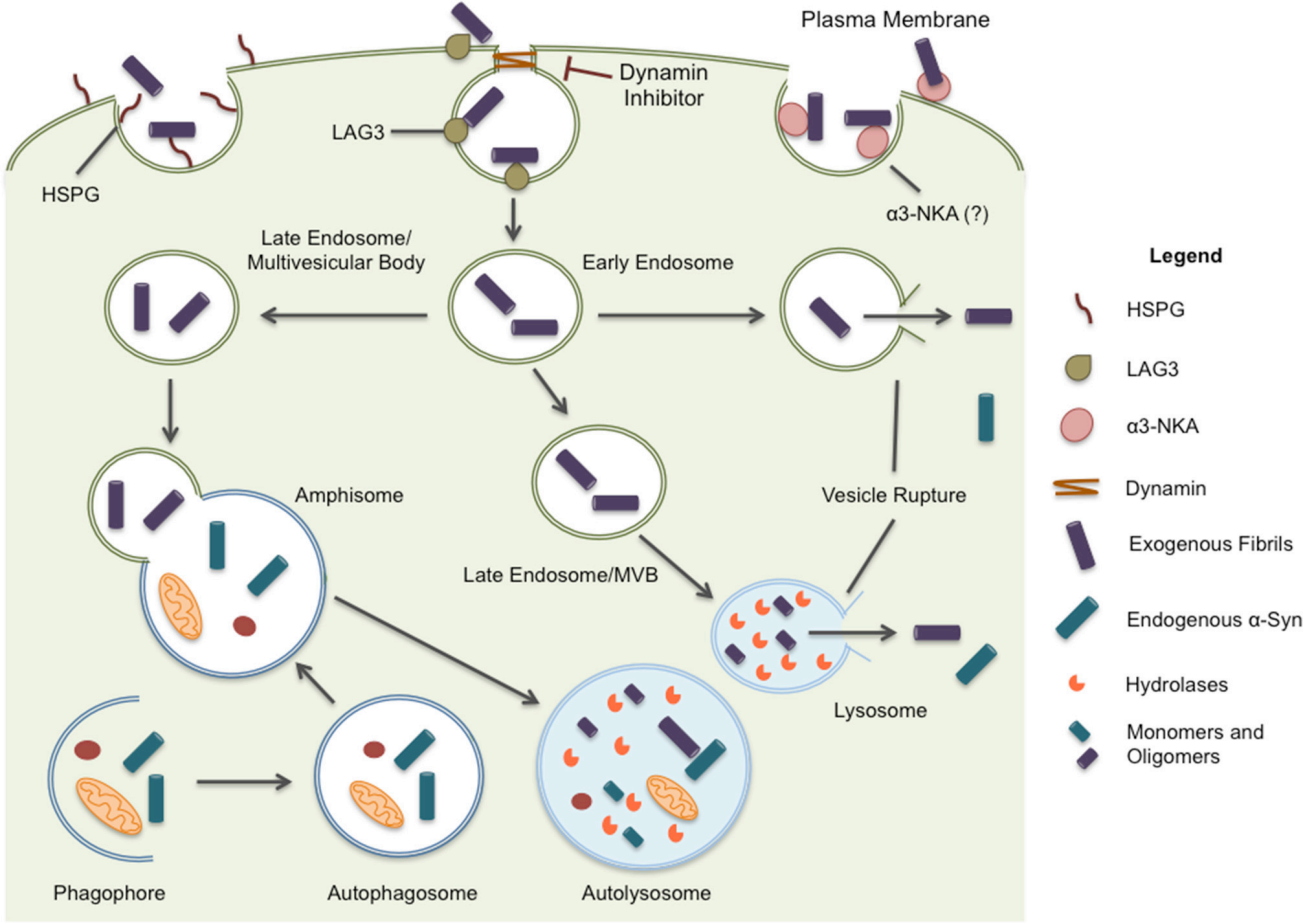
α-syn is believed to enter the cell through endocytosis and three potential receptors, HSPG, LAG3, and α3-NKA, have been implicated in the internalization of α-syn. Once inside the cell, the protein may undertake multiple pathways. Endocytic vesicles are directed to the autophagic system for degradation. In some circumstances, endocytic vesicles may fuse with autophagosomes to create hybrid structures, referred to as amphisomes. Both endosomes and amphisomes merge with lysosomes where their internal contents are degraded by hydrolases. Alternatively, it has been proposed α-syn is capable of inducing vesicle rupture in endosomes and lysosomes resulting in the release of internalized protein into the cytoplasm. This provides a unique opportunity for intracellular protein and internalized exogenous fibrils to interact. Whether the interaction occurs within merged vesicles or in the cytoplasm following expulsion from endocytic organelles, these rupture events likely allow for the seeding and propagation of misfolding protein in a disease model.

Different mechanisms for internalization have been proposed including endocytosis, micropinocytosis, and cell surface protein-mediated uptake (Figure 1). Of these, endocytosis has been the most studied (see Table 1 for a summary of relevant studies on endocytosis). Briefly, dynamin facilitates the budding of endocytic vesicles from the plasma membrane, which merge with early endosomes. Cargo within the endosomal structures can recycle back to the plasma membrane, be released through exosomes, or fuse with lysosomes for degradation (Huotari and Helenius, 2011). Inhibiting endocytosis, either by maintaining cells at a lower, non-permissive temperature or with a dominant negative dynamin-1 mutant, substantially reduces fibrils uptake in all cell lines tested, including primary neurons (Lee et al., 2008a; Abounit et al., 2016). The same effect is evident in the presence of dynamin inhibitors such as Dynasore (Samuel et al., 2016; Sacino et al., 2017). Also, in accord with the capture of misfolded α-syn oligomers/fibrils by endocytic vesicles, an upper limit of ~50 nm was observed for α-syn fragments, presumably defined by vesicle lumen capacity (Tarutani et al., 2016). Moreover, fibril entry appears to saturate at high fibril concentrations indicating an upper limit to the uptake pathway (Karpowicz et al., 2017). These features are all consistent with endocytosis as the primary mechanism of α-syn internalization. In addition, internalized fibrils co-localize with markers of the endocytic pathway (EEA1, Rab5) and are rapidly acidified within hours of treatment before finally co-localizing with markers of late endosomal and lysosomal compartments (Lamp-1) (Lee et al., 2008a; Konno et al., 2012; Karpowicz et al., 2017).

**Table 1:** α-Syn aggregates and endocytosis *in vitro*.

**α-Syn Species**	**Cell Type**	**Treatment**	**References**
Monomers	H19-7 Cells		Sung et al., 2001
Fibrils	Microglia		Zhang et al., 2005
Fibrils	Microglia		Liu et al., 2007
Fibrils	SH-SY5Y, Primary Neurons	DN-Dynamin	Lee et al., 2008a
Monomers, Fibrils	BV-2 Cells		Park et al., 2008
Fibrils	Neurons, Astrocytes, and Microglia	DN-Dynamin	Lee et al., 2008b
Monomers	BV-2 cells	Lipid Raft Inhibitor	Park et al., 2009
Oligomers, Conditioned Media	MCNSC, Primary Neurons	DN-Dynamin	Desplats et al., 2009
Conditioned Media	SH-SY5Y, Primary Neurons, Astrocytes	DN-Dynamin	Lee et al., 2010
Fibrils	Primary Neurons	4C Wheat Germ Agglutinin (WGA)	Volpicelli-Daley et al., 2011
Oligomers, Exosomes	H4 Neuroglioma, Primary Neurons		Danzer et al., 2012
Monomers, Oligomers	SH-SY5Y, KG1C Oligodendroglial Cells, Primary Neurons	WGA, Sertraline (Dynamin Inhibitor), DN-Dynamin, siRNA	Konno et al., 2012
Monomers, Oligomers	SH-SY5Y	Nedd4	Sugeno et al., 2014
Fibrils	SH-SY5Y		Aulić et al., 2014
Fibrils	Primary Neurons	Immunotherapy	Tran et al., 2014
Fibrils	Primary Neurons	α-Syn KO Neurons	Volpicelli-daley et al., 2014
Monomers, Oligomers, Fibrils	Oligodendrocytes	Dynasore	Reyes et al., 2014
Fibrils	MSCs, SH-SY5Y	Dynasore, Pitstop	Oh et al., 2016
Fibrils	Primary Neurons		Brahic et al., 2016
Fibrils	CAD Cells, Primary Neurons	DN-Dynamin	Abounit et al., 2016
Fibrils	SH-SY5Y, Dopaminergic Neurons	Dynasore	Samuel et al., 2016
Oligomers, Exosomes Associated Oligomers	Mixed Glial Cultures		Bliederhaeuser et al., 2016
Fibrils	Primary Neuron-Glia Culture	Dynasore	Sacino et al., 2017
Monomers, Fibrils	Primary Neurons		Mao et al., 2016
Aggregates from PD brains	Primary Neuron-Glia culture		Cavaliere et al., 2017
Oligomers, Exosomes Associated Oligomers	H4 Neuroglioma Cell, CHO	Chlorpromazine (Clathrin Inhibitor) Nystatin (Caveolin Inhibitor) Cytocholasin D (Macropinocytosis Inhibitor), HSPG-KO Cells	Delenclos et al., 2017
Oligomers, Fibrils	Embryonic Cortical Stem Cells, Astrocytes (75%), Neurons (20%) and Oligodendrocytes (5%)		Lindström et al., 2017
Fibrils, Oligomers	SH-SY5Y, iPSC		Flavin et al., 2017
Fibrils	Primary Neurons, Astrocytes		Loria et al., 2017
Fibrils	Primary Neurons	Trypan Blue, Heparin	Karpowicz et al., 2017
Monomers, Oligomers, Fibrils	B103 Cells, Oligodendrocytic MO3.13 Cells and Murine Microglial BV-2 Cells, Rat Glioma C6 Cells	Heparin, Chondroitin Sulfate	Ihse et al., 2017

On the other hand, heparan sulfate proteoglycan (HSPGs) mediated-uptake of α-syn fibrils by micropinocytosis has also been shown. In non-neuronal cell lines, α-syn fibrils bind to heparan sulfate chains in the plasma membrane prior to internalization (Holmes et al., 2013). The interaction between the sulfated glycosaminoglycan (GAG) chains and α-syn fibrils likely occurs through contact between the negatively charged groups in the GAG chains and positively charged amino acids in the amyloid protein. Soluble heparin in culture media can competitively inhibit cell surface binding and uptake of α-syn fibrils into primary neurons (Karpowicz et al., 2017). Internalization through this mechanism seems to be dependent on the degree of α-syn aggregation as well as cell type. HSPG-mediated uptake is more common in non-immune cells of the brain such as oligodendrocytes and neurons, while astrocytes and microglia seem to employ additional mechanisms for internalization (Ihse et al., 2017).

In conjunction with HSPGs, other cell surface receptors for α-syn fibrils have been identified. Lymphocyte activation gene-3 (LAG-3), a member of the immunoglobulin superfamily of receptors, binds to α-syn fibrils and triggers endocytosis into neurons (Mao et al., 2016). Blockade or knockdown of LAG-3 in neuronal cultures or animals diminished the transmission of α-synuclein between neurons and reduced the accumulation of fibrils. However, the inhibition was incomplete, and the same study also found neurexin 1b, and Aβ precursor-like protein 1 (APLP1) act as putative receptors, suggesting that multiple cell surface molecules may contribute to the entry of α-syn fibrils into cells. Another study used a proteomic screen to identify several membrane interactors of α-syn oligomers and fibrils; in addition to neurexin 1b, the α3-subunit of Na^+^/K^+^-ATPase (NKA) was also detected as a potential cell surface interactor with α-syn fibrils (Shrivastava et al., 2015). Clustering of α-syn at the membrane induced the redistribution of α3-NKA, and although the role of α3-NKA in α-syn endocytosis was not investigated, the interaction reduced its ability to pump out Na^+^ from neurons. While many cell-surface receptors have been proposed, it remains unclear whether different α-syn assemblies, namely oligomers, fibrils, or exosome-packed, are internalized via distinct receptors or endocytic mechanisms in neurons.

## α-synuclein trafficking and proteostasis

Following internalization, α-syn fibrils are trafficked to late endosomal compartments and lysosomes (Lee et al., 2008a; Abounit et al., 2016; Karpowicz et al., 2017). Differences in uptake kinetics and fibril degradation between studies may be related to differences in culture conditions and the types of α-syn assemblies used (Loria et al., 2017; Sacino et al., 2017). Once internalized, the movement of endocytosed α-syn fibrils by primary neurons matches the kinetics of slow component b of axonal transport, and although bidirectional, retrograde transport predominates over the anterograde (Brahic et al., 2016). Whether internalized fibrils are transported as naked assemblies or in endocytic compartments remains to be determined; however, the latter seems most likely.

Lysosomal inhibition has been shown to cause an accumulation of α-syn, suggesting the autophagy/lysosomal pathway is involved in the clearance of oligomeric and fibrillar species of α-syn (Lee et al., 2004; Vogiatzi et al., 2008; Sacino et al., 2017). Macroautophagy, chaperone-mediated autophagy (CMA), and microautophagy direct intracellular constituents to the lysosome, and all of these processes could be involved in α-syn degradation. The delivery of α-syn to the lysosome is mediated by both chaperone-mediated autophagy (CMA) and macroautophagy. CMA depends on the recognition of CMA-targeting motifs by Hsc70, co-chaperone complexes, and Lamp-2A; while macroautophagy is regulated by multiple autophagy-related gene (Atg) products that mediate a multistep process to envelope cytosolic components and organelles into double-membrane vesicles that merge with lysosomes (Cuervo et al., 2004; Lamb et al., 2013; Jackson and Hewitt, 2016).

Monomeric and dimeric α-syn species are degraded by CMA in isolated lysosomes *in vivo*, and familial α-syn mutants (A30P and A53T) have been shown to inhibit CMA (Cuervo et al., 2004; Mak et al., 2010). In cultured cells and primary neurons, downregulation of Lamp-2A led to an increase in insoluble α-syn, suggesting degradation through CMA does not merely pertain to monomeric species of α-syn (Vogiatzi et al., 2008). Furthermore, *in vitro* and α-syn transgenic animal models of aggregation showed α-syn was found in Lamp-2A- positive inclusions (Klucken et al., 2012). *In vivo*, overexpression of Lamp-2A led to a decrease in α-syn turnover and selective dampening of α-syn neurotoxicity, highlighting the importance of CMA in α-syn degradation (Xilouri et al., 2013).

Other studies suggest macroautophagy also contributes to the degradation of α-syn aggregates, in large part because α-syn has been shown to interact with autophagic markers in cell models (Crews et al., 2010; Tanik et al., 2013). α-Syn aggregates are predominantly co-localized with LC3 and p62 in neurons, suggesting an accumulation at the autophagosome stage of autophagy (Tanik et al., 2013). Similarly, acute lentiviral-mediated α-syn overexpression in rat neuroblastoma cells causes a build-up of autophagic vesicles containing α-syn. The addition of Beclin-1 ameliorated the toxic effects by enhancing autophagy, reducing the build-up of α-syn in a transgenic mouse model, and improving the neuronal deficits induced by α-syn overexpression (Spencer et al., 2009).

In addition, inhibition of macroautophagy by 3-methyladenine (3-MA) increases both soluble and insoluble α-syn levels in non-neuronal cells, and elevates the levels of endogenous α-syn in rat cortical and ventral midbrain dopaminergic neurons (Vogiatzi et al., 2008). Similarly, pharmacological or molecular inhibition of macroautophagy promotes the accumulation of A53T α-syn oligomers in neuroblastoma cells (Yu et al., 2009). However, one study examined the relationship between α-syn overexpression and lysosomal inhibition and found bafilomycin A1, but not 3-MA, resulted in an accumulation of insoluble α-syn with a concomitant increase in α-syn puncta over aggregates. This was modeled in both neuronal cultures and transgenic mice and the authors concluded that a lysosomal pathway independent of macroautophagy is likely responsible for the degradation of insoluble α-syn (Klucken et al., 2012). It has also been shown that induction of non-neuronal and neuronal cells with preformed fibrils leads to intracellular α-syn aggregates, which are poorly degraded by macroautophagy (Tanik et al., 2013).

Hence, macroautophagic degradation of α-syn may be dependent on its conformation and post-translational modifications, although it is still not completely understood which pathway is preferred by neurons for degrading oligomeric and fibrillar α-syn species. Oligomeric intermediate species seem to be susceptible to clearance by CMA and macroautophagy, whereas mature fibrillar inclusions are not. Some studies have noted α-syn secretion is enhanced by macroautophagic/lysosomal inhibition; this suggests exocytosis could be a central mechanism for the clearance of α-syn aggregates (discussed below) (Jang et al., 2010; Danzer et al., 2012; Lee et al., 2013; Poehler et al., 2014).

Three recent reports have studied the fate of internalized α-syn fibrils in neuronal cells under physiological conditions (i.e., non-overexpressed α-syn). In 2017, Sacino et al., showed that exogenously added α-syn fibrils were progressively degraded by mixed neuronal-glial cultures with a half-life of 3–5 days. Lysosomal inhibition in these cells resulted in the accumulation of α-syn fibrils in vesicles. Another study, using either neuronal or astrocytic cultures, showed a progressive decrease of full-length α-syn fibrils over time accompanied by an increase in cleaved products in the neuronal cultures. Conversely, astrocytes degraded both full-length and cleaved fragments more efficiently, suggesting that fibrils degrade more slowly in neuronal cultures (half-life of 6-9 days) vs. astrocytic cultures (Loria et al., 2017). In accordance with these results, other studies have shown minimal degradation of exogenously added α-syn fibrils in neuronal cultures (Karpowicz et al., 2017; Loria et al., 2017). Understanding the differences in the clearance of α-syn aggregates in different types of brain cells will have important implications for understanding the mechanism of seeding and spreading of aggregates; especially as these studies support the hypothesis that astrocytes play a neuroprotective role against the propagation of α-syn pathology.

## Intracellular interaction with endogenous α-synuclein

An appealing hypothesis suggests the spreading of α-syn pathology in PD is a result of permissive templating, whereby misfolded species of α-syn interact with normal, intracellular α-syn causing a conformational change. This is consistent with the mechanisms in prion disorders whereby healthy prion protein (PrP^C^) is converted into its misfolded conformer (PrP^Sc^) (Colby and Prusiner, 2011). The inherent self-propagating nature of these interactions within and between cells is therefore thought to underlie the progressive aspect of the PD pathology.

Co-localization of internalized α-syn fibrils and intracellular α-syn protein has been shown in both neuronal and non-neuronal cell lines, supporting the idea that exogenous α-syn fibrils act as a nucleating seed to recruit intracellular α-syn into larger assemblies (Luk et al., 2009; Waxman and Giasson, 2010; Volpicelli-Daley et al., 2011). These aggregates stain for markers relevant to Lewy body pathology, such as α-syn phosphorylated at the serine 129 position (pSer129) and Thioflavin S (Desplats et al., 2009; Luk et al., 2009; Volpicelli-Daley et al., 2011). There is now sufficient evidence to suggest that exogenous α-syn can imprint its intrinsic structural characteristics onto endogenous α-syn creating distinct strains of misfolded protein (Bousset et al., 2013; Watts et al., 2013; Peelaerts et al., 2015). Importantly, intracellular aggregation does not occur upon exposure to monomeric α-syn or soluble fibrils (Waxman and Giasson, 2008; Luk et al., 2009). Furthermore, the finding that α-syn-deficient neurons are resistant to exogenous aggregates suggests that endogenous recruitment is imperative for pathology (Volpicelli-Daley et al., 2011). While numerous studies have described the parameters involved in seeding between exogenous and intracellular α-syn, the location of these interactions requires further clarification.

Because endocytosed α-syn fibrils are likely encapsulated in the lumen of endocytic vesicles and downstream compartments, there is an obvious question as to how the lumenal protein might escape the endocytic compartment to interact with intracellular cytoplasmic α-syn. Propagation by prion-like means predicts that extracellular α-syn must interact directly with endogenous protein. One possibility is that misfolded α-syn is able to disrupt membranes and exit the endocytic pathway, analogous to viral entry into cells with the aid of an amphipathic protein that ruptures endocytic vesicles (Wiethoff et al., 2005). The process can be measured with cytoplasmically expressed galectin-3 (Gal-3), which relocalizes to ruptured vesicles due to its affinity for β-galactoside sugars on the lumenal membrane (Paz et al., 2010; Maier et al., 2012). Using this assay, treatment of mCherry-Gal-3 expressing cells with fibrillar, but not monomeric α-syn, caused Gal-3 fluorescence to redistribute from the cytoplasm to intracellular vesicles, consistent with α-syn-mediated vesicle rupture (Freeman et al., 2013; Samuel et al., 2016; Flavin et al., 2017). Co-localized α-syn and mCherry-Gal-3 were detected with early endosome and lysosomal markers providing a mechanism by which endocytosed misfolded α-syn can directly interact with intracellular α-syn in the cytosol after escaping from ruptured vesicles.

The prevailing evidence indicates extracellular fibrils enter cells and are subsequently directed to the endo-lysosomal pathway (Lee et al., 2008a; Apetri et al., 2016; Flavin et al., 2017; Karpowicz et al., 2017). Exogenous fibrils, labeled with a pH sensitive dye, were applied to neurons and by four hours over 50% of fibrils were located within acidic vesicles; these levels rose and were persistent up to 7 days. Further investigation showed these fibrils also co-localized with the lysosomal marker Lamp-1 (Karpowicz et al., 2017). Conversely, overexpression and endogenous expression models have demonstrated co-localization between PSer129 α-syn and autophagic markers such as LC3 and p62 following exposure to fibrils (Tanik et al., 2013). Therefore, in addition to vesicle rupture by endocytosed α-syn fibrils, another point of interaction between exogenous and intracellular α-syn may be within vesicles along the autophagic pathway. Evidently, there are many opportunities for seeding events to occur and exert their toxic effects on the cell. Whether the predicted cytosolic or lumenal α-syn interactions occur independently, in parallel, or in sequence remains to be determined.

## Export and transfer of α-synuclein fibrils

Conventionally secreted proteins are co-translationally transported into the endoplasmic reticulum (ER) and trafficked along the exocytic pathway to the cell surface (Viotti, 2016). There is general agreement that α-syn is released through an unconventional secretion pathway (Lee et al., 2005, 2016; Jang et al., 2010), which enables proteins lacking a signal peptide to reach the plasma membrane bypassing the Golgi (Rabouille, 2017). Several studies have identified monomeric and multimeric α-syn in extracellular vesicles, although there remains debate regarding the precise species of α-syn contained within these structures. Furthermore, the source of the secretory organelle has yet to be identified (Lee et al., 2005; Jang et al., 2010; Danzer et al., 2011; Brahic et al., 2016).

**Figure 2:**
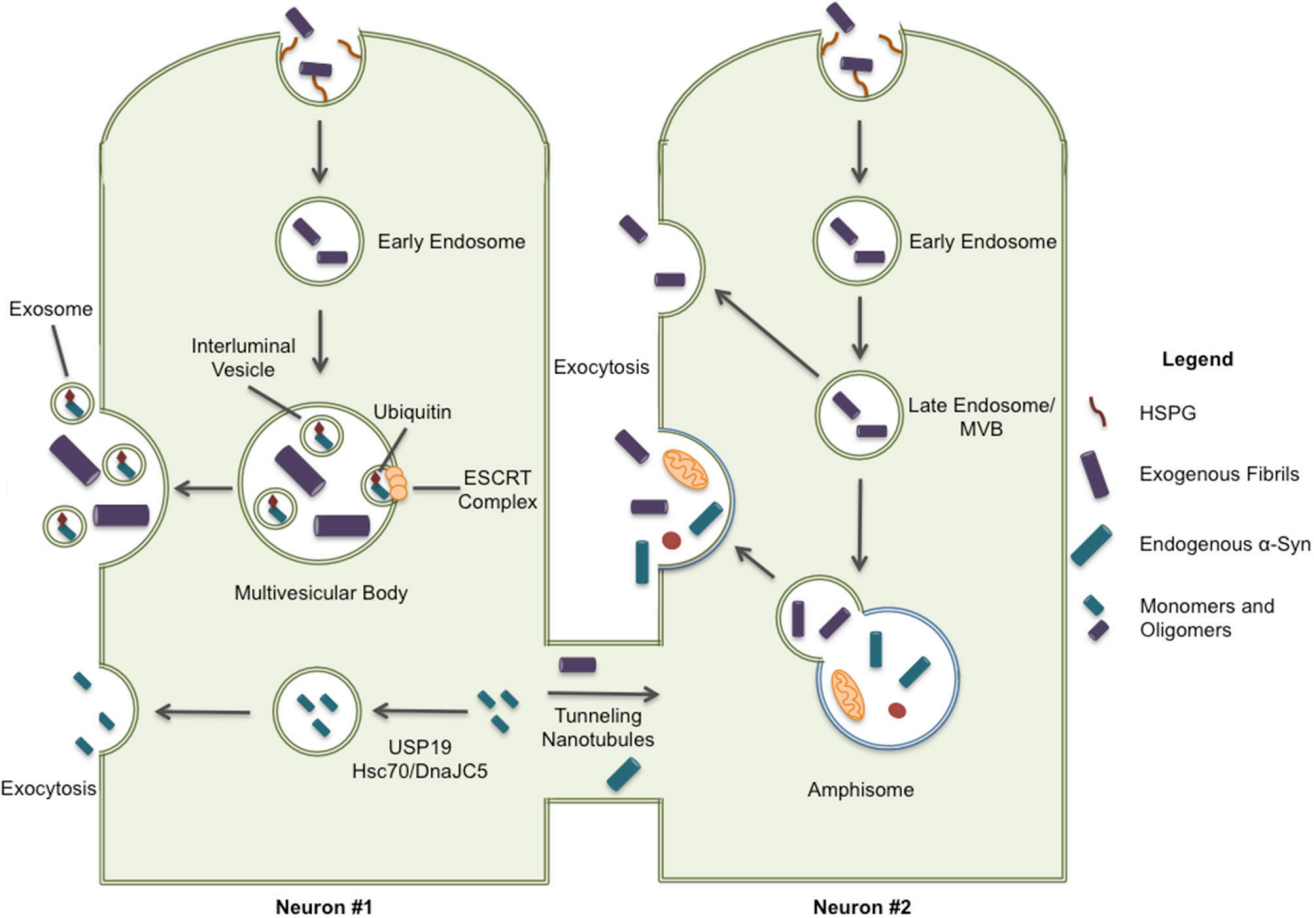
Some pathways have been implicated in the secretion of α-syn protein. ESCRT-mediated import of intracellular α-syn to multivesicular bodies can result in the excretion of α-syn through exosomal release; although, it should be noted this form of secretion is only associated with monomeric and oligomeric forms of α-syn. Cytoplasmic α-syn is also recruited to Rab9a-positive vesicles through chaperone-mediated pathways involving USP19 and Hsc70/DnaJC5 leading to exocytosis. Lastly, intracellular α-syn is secreted through tunneling nanotubules (TNTs) to neighboring cells providing a direct path for the spreading of pathology. When internalized proteins are not immediately directed to protein degradation systems, they may also be released through exocytosis. This exocytic process can occur directly from late endosome/multivesicular bodies and, more recently, release from secretory autophagic vesicles has also been described.

Two recent studies shed some light on the possible mechanisms of fibrillar α-syn secretion. In 2016, Lee et al. identified a secretion pathway where misfolded cytoplasmic proteins are released into the extracellular space by way of a cellular stress response (Lee et al., 2016). USP19, an ER-associated deubiquitylase, acts as a chaperone and escorts the waste into a newly described export pathway, particularly when the proteasome becomes overwhelmed (Figure 2). They found that α-syn, but not tau, was secreted in a USP19-dependent fashion. More specifically, USP19 mediated the recruitment of misfolded proteins to Rab9-positive endosomes prior to secretion. Currently, it is unclear if neurons follow this mechanism for secretion. Moreover, because substrates in this pathway must translocate from the cytoplasm into the ER, it seems unlikely that large aggregates could enter this secretion pathway. A second study showed that the chaperone complex Hsc70/DnaJC5 binds to different proteins connected with neurodegenerative diseases, including tau and α-syn, and facilitates their removal from neurons (Figure 2; Fontaine et al., 2016). SNAP-23 has been implicated in this process as it's knockdown in HEK293 and neuroblastoma cells decreased DnaJC5-mediated release of tau and α-syn (Fontaine et al., 2016). How, and where in the cell, the protein cargos enter this export pathway is unknown. In addition, the conformation of α-syn secreted by this pathway has yet to be determined.

Whether aggregated α-syn is secreted by neurons through any of these mechanisms still needs to be demonstrated. In addition, the specific compartment used to exit the cell has not been identified. Organelles like late endosomes/multivesicular bodies (MVBs), autophagosomes, and amphisomes constitute attractive candidates for the release of misfolded proteins, such as α-syn, through exocytosis. These organelles are known to undergo physiological exocytosis (Ponpuak et al., 2015), a process that could be enhanced under stressful (lysosomal inhibition) or pathological conditions (PD genetic risk factors) (Tsunemi et al., 2014). All possible secretion pathways described previously suggest a vesicle-derived exocytosis mechanism. ESCRT-mediated import of ubiquitinated cargo into MVBs, or the direct uptake of cytosolic cargo into autophagosomes by selective autophagy, could explain a route of entry for α-syn fibrils into the lumen of these compartments (Hasegawa et al., 2011; Sugeno et al., 2014). Note that secretory autophagosomes may fuse with MVBs to generate amphisomes before fusion with lysosome or the plasma membrane (Figure 2). On the other hand, microautophagy, where cargo is imported into late endosomes and the degradative process occurs in late endosomes/MVBs, could also mechanistically explain the presence of α-syn fibrils in these organelles (Sahu et al., 2012; Tekirdag and Cuervo, 2017).

Some misfolded proteins, including tau, reportedly leave the cell via vesicular packages called exosomes that are released by the fusion of MVBs with the plasma membrane (Figure 2; Saman et al., 2012). However, it has been reported that export via exosomes tends to favor normal, folded proteins (Lo Cicero et al., 2015). The exosomal-association of fibrillar α-syn is still under debate and may depend on the cellular model used as some groups find little or no association of α-syn with secreted exosomes (Ejlerskov et al., 2013; Fernandes et al., 2016). Thus far oligomers, but not fibrils, have been located in exosomes (Danzer et al., 2012).

Lastly, tunneling nanotubules (TNT) have also been proposed as a mechanism for α-syn transfer between cells (Figure 2). TNTs are actin-based membrane channels that connect cells (Dieriks et al., 2017). Recently, it has been shown that α-syn can move between cells through TNTs and appears to be directed to the lysosome in recipient cells (Abounit et al., 2016). TNT transfer has been observed in primary neurons as well as pericytes (Abounit et al., 2016; Dieriks et al., 2017).

## Final remarks

Our understanding of α-syn pathobiology has advanced rapidly in recent years from its widely replicated behavior as a small unfolded protein in nerve terminals that self-aggregates into fibrils resembling the pathological assemblies observed in Lewy bodies. The recognition that these longer structures are not mere endpoints of a disease process, but intermediate and transferable agents of the disease, is leading to potential therapies that were thought unlikely to succeed only a few years ago, such as α-syn gene silencing and vaccines to clear extracellular α-syn. Nevertheless, there remain many unresolved questions as to how α-syn misfolding and propagation is exacerbated by age-related changes to multifactorial physiological processes, including immune response, inflammation, oxidative stress, and protein degradation, which could conceivably be targeted to mitigate neurodegenerative processes before their clinical manifestation.

## Author contributions

All authors listed have made a substantial, direct and intellectual contribution to the work, and approved it for publication.

### Conflict of interest statement

The authors declare that the research was conducted in the absence of any commercial or financial relationships that could be construed as a potential conflict of interest. The reviewer EVM and handling Editor declared their shared affiliation.
